# Laser lithotripsy for pancreatolithiasis in pancreas divisum

**DOI:** 10.1055/a-2285-2491

**Published:** 2024-04-09

**Authors:** Rohit Gupta, Sugata Narayan Biswas

**Affiliations:** 1 Department of Gastroenterology, Foundation for Research and Education in Gastroenterology and Endoscopy, Prayagraj, India; 2 Department of Gastroenterology, Moti Lal Nehru Medical College, Prayagraj, India

Pancreas divisum is the most common congenital anomaly of the pancreas with a reported prevalence of 6%–10% 1 2 . Pancreas divisum may predispose to recurrent acute pancreatitis and its complications, and eventually may result in chronic pancreatitis in a subset of patients. In the setting of chronic pancreatitis due to pancreas divisum, therapeutic intervention in the form of minor papilla sphincterotomy, balloon dilation of the papillary orifice, and transpapillary dorsal duct stenting may be indicated in patients with abdominal pain refractory to medical therapy, presence of stones or stricture, and/or a dilated dorsal pancreatic duct. Laser lithotripsy in the management of pancreatolithiasis in patients with pancreas divisum has been rarely reported 3 .

A 32-year-old man, previously diagnosed with pancreas divisum leading to chronic pancreatitis with pancreatolithiasis, was referred for endotherapy because of persistent pain refractory to medical therapy. Endoscopic retrograde pancreatography was performed ( Video 1 ) by cannulating the minor papilla and using a triple-lumen sphincterotome (Clever Cut 3 V; Olympus, Tokyo, Japan) and a 0.025-inch guidewire (Jagwire; Boston Scientific, Marlborough, Massachusetts, USA). The pancreatogram revealed a dilated pancreatic duct with a filling defect in the proximal part near the head of the pancreas. Using the sphincterotome, papillotomy was performed, followed by balloon papilloplasty to an 8-mm diameter. Intraductal balloon trawl failed to deliver the stone, and two 7 Fr × 8 cm straight pancreatic stents were placed to relieve intraductal hypertension, facilitate pancreatic drainage, and allow smooth passage of the pancreatoscope during future endotherapy.

Endoscopic retrograde pancreatography with minor papilla cannulation, papilloplasty, and stenting followed by pancreatoscopy and laser lithotripsy of pancreatic ductal stone in a case of pancreas divisum with chronic pancreatitis.Video 1

Definitive management options in the form of extracorporeal shock wave lithotripsy, electrohydraulic lithotripsy, laser lithotripsy, and surgery were discussed with the patient. The patient opted for endotherapy over surgery. As only laser lithotripsy is available at our center, it was offered to the patient. Repeat endoscopic retrograde pancreatography was performed a week later. The stents were removed, and balloon papilloplasty was performed followed by advancement of a pancreatoscope (Spyglass DS; Boston Scientific) through the duct. A white stone was visualized in the pancreatic duct, which was fragmented using holmium laser lithotripsy (10 W for 2 J at 5 Hz). Multiple balloon sweeps were made to remove the stone fragments. Repeat pancreatoscopy documented complete clearance of the pancreatic duct.

The patient was admitted for observation and did well without any adverse events. At follow-up 6 months later, he remained pain free, while his abdominal ultrasound did not reveal any recurrence of pancreatolithiasis.

Endoscopy_UCTN_Code_CPL_1AH_2AH

## Notes

Endoscopy E-Videos https://eref.thieme.de/e-videos
*E-Videos* is an open access online section of the journal *Endoscopy* , reporting on interesting cases and new techniques in gastroenterological endoscopy. All papers include a high-quality video and are published with a Creative Commons CC-BY license. Endoscopy E-Videos qualify for HINARI discounts and waivers and eligibility is automatically checked during the submission process. We grant 100% waivers to articles whose corresponding authors are based in Group A countries and 50% waivers to those who are based in Group B countries as classified by Research4Life (see: https://www.research4life.org/access/eligibility/ ). This section has its own submission website at https://mc.manuscriptcentral.com/e-videos .
